# Use of an Electronic Feeds Calorie Calculator in the Pediatric Intensive Care Unit

**DOI:** 10.1097/pq9.0000000000000249

**Published:** 2020-01-12

**Authors:** Qian Wen Sng, Chengsi Ong, Su Ling Linda Ang, Angela Hui Ping Kirk, Jan Hau Lee

**Affiliations:** * Division of Nursing, KK Women’s and Children’s Hospital, Singapore; † Children’s Intensive Care Unit, Department of Pediatric Subspecialties, KK Women’s and Children’s Hospital, Singapore; ‡ Department of Nutrition and Dietetics, KK Women’s and Children’s Hospital, Singapore; § Duke-NUS Medical School, Singapore.

## Abstract

Strategies to improve nutritional management are associated with better outcomes in pediatric intensive care units. We implemented a calorie-based protocol that integrated an electronic feeds calculator and stepwise feeds increment algorithm.

**Methods:**

Using a pretest–posttest design, we compared the effectiveness of the calorie-based protocol with an existing fluid-based protocol in a quality improvement project. The main outcome measure was the proportion of patients prescribed with the appropriate amount of calories (defined as 90%−110% of calculated energy requirements). Nurses were surveyed on their satisfaction with the new calorie-based protocol. We compared consecutive patients enrolled in the calorie-based protocol over 21 months with retrospective data of patients in the fluid-based protocol. *χ*^2^ and Mann–Whitney U tests were used to compare categorical and continuous variables, respectively.

**Results:**

We enrolled 75 and 92 patients in the fluid-based (pre) and calorie-based (post) protocols, respectively. Both groups did not differ in their age, reasons for pediatric intensive care units admissions, length of stay, duration of mechanical ventilation, and risks of mortality. The frequency of appropriate feeds prescription increased (16.0% versus 33.7%, *P* = 0.002). The new protocol significantly reduced the time from protocol initiation to full feeds (median: 18.0 hours, interquartile range = 18.0−27.5 versus median: 12.8 hours, interquartile range = 12.0−16.0, *P* < 0.001). The satisfaction surveys (n = 63) revealed favorable nursing perceptions.

**Conclusions:**

The use of a calorie-based protocol with an electronic calculator led to an improvement in the accuracy of the prescribed feeds and the time required to attain full enteral feeding. Nursing perceptions regarding the protocol were positive.

## Abstract 2

Supplemental Digital Content is available in the text.

## INTRODUCTION

Malnutrition is common among patients admitted to pediatric intensive care units (PICU).^1–3^ Among mechanically ventilated patients, underweight was associated with higher mortality and nosocomial infections, whereas overweight was associated with a prolonged hospital length of stay.^3^ The lack of enteral achievement of caloric goals is associated with higher mortality rates.^4,5^ Enteral nutrition (EN) protocols can be used to deliver more EN and commenced feeding earlier in intensive care units (ICUs).^6,7^ EN protocols automate and standardize EN delivery. Bedside nurses can initiate feeds, monitor response, and progress, or halt feeds according to an algorithm.

In our PICU, we formed a multidisciplinary team to revamp the former nurse-led fluid-based feeding protocol. This fluid-based protocol was implemented in 2013 to provide clear guidelines on the initiation and gradation of enteral feedings based on clinical assessments. However, a review of the process and patients’ clinical data revealed 3 main areas for improvement: (1) fluid-based prescription of milk feeds; (2) nursing work time spent on manual calculation; and (3) slow, incremental increase of feeds. In the fluid-based feeding protocol, physicians prescribed patients’ milk feeds based on total fluid requirements without accounting for caloric requirements during critical illness. Ideally, feedings should be tailored to individual patient’s caloric requirements to prevent over- and underfeeding.^8^ However, dietitians are not always stationed in the PICU. Furthermore, the fluid-based feeding protocol required nurses to manually calculate feeding volume and corresponding intravenous drip at each stage of feeding increase. This requirement was time-consuming and error prone. Lastly, the protocol took longer to achieve full enteral feeding than recommended by international nutrition guidelines.^8^

**Fig. 1.**
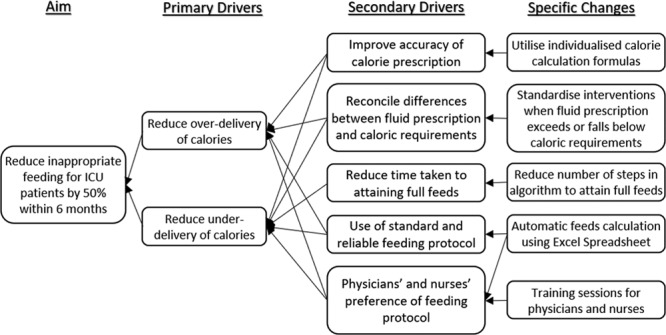
Key Drivers’ diagram.

Thus, to improve the quality of nutritional management in the PICU, we designed and introduced an electronic feed calculator using Microsoft Excel 2013 (Microsoft, Redmond, WA, USA) to improve the appropriateness of initial feed prescription in 2016 (Fig. 1). This study aims to evaluate the impact of a customized feed calculator to a nurse-led feeding protocol. We also aimed to determine nurses’ perceptions of the prescription of feeds using the new calculator.

## METHODS

### Setting and Participants

We conducted a quality improvement project using a pretest–posttest design in a 16-bed multidisciplinary PICU in a tertiary women’s and children’s hospital in Singapore. The Centralized Institutional Review Board approved this study with a waiver for informed consent. Data of consecutive patients prospectively collected over 21 months (September 2016 to May 2018) were compared with the cohort of patients enrolled in the fluid-based protocol (February 2013 to April 2016). The medical team initiated the EN protocol with appropriate input from the nurses. We fed patients aged <1-year-old every 3 hours and older patients every 4 hours. Dietitians were consulted if there were concerns about failure to thrive or inappropriate feeds. Both protocols had the same exclusion criteria: oral feeding, admission weight <2 kg, postgastrointestinal surgery, central cooling, impending intubation or extubation, septic shock, postcardiac surgery, and recently suspected or confirmed necrotizing enterocolitis. For patients with multiple PICU admissions, we only included the first admission for this study.

### Fluid-based Protocol (Preintervention)

Physicians routinely prescribed maintenance fluid allowance using the Holliday-Segar method,^9^ with additional fluid restrictions as clinically indicated. The Holliday-Segar method estimates maintenance fluid in the following manner:

For children between 0 and 10 kg, 100 ml/kg,
10 to 20 kg, 1000 ml + 50 ml/kg for each kg over 10 kg,
>20 kg, 1500 ml + 20 ml/kg for each kg over 20 kg.

In the fluid-based protocol, we converted the maintenance fluid allowance into feeding goals. Nurses derived feeding goals and maintenance drip volumes using manual calculation on pen and paper. The feeding protocol begins with the first feeding of 25% of the derived feeding goal. Nurses assessed feeding intolerance and determined the subsequent feeding volume according to the protocol. Full feeding (defined as 100% of feeding goal) was achieved in 7 steps: 25% for 2 feedings, 50% for 2 feedings, 75% for 2 feedings, and lastly 100% of feeding goal. Feeds were further adjusted by a dietitian after 1–3 days if necessary.

### Interventions

The team designed the nurse-led calorie-based protocol with the primary aim of establishing appropriate and individualized initial feeding goals (see Table, Supplemental Digital Content 1, which compares calories provided by fluid-based and calorie-based protocols, http://links.lww.com/PQ9/A154). Feeding goals were calculated to meet estimated energy requirements calculated using the Schofield equations, with adjustments according to the patient’s age, sex, weight, and clinical status (eg, intubation status) (see Table, Supplemental Digital Content 2, which presents the Schofield equations, http://links.lww.com/PQ9/A154).^10^ Also, the team accelerated the progression of feeds in the new EN algorithm in 5 steps: 25% for 2 feedings, 50% for 1 feeding, 75% for 1 feeding, and lastly, 100% of feeding goal (see Figure, Supplemental Digital Content 3, which displays the feeding algorithm for calorie-based protocol, http://links.lww.com/PQ9/A154).

Due to the use of the varying types of feeds and multiple calculations required to reconcile energy and fluid needs, we created a novel feed calculator using Microsoft Excel 2013 (Microsoft, Redmond, WA, USA) to initiate and advance feeds (see Figure, Supplemental Digital Content 4, which illustrates the Electronic Feed Calculator, http://links.lww.com/PQ9/A154). To use the calculator, nurses entered the following information: patient’s weight, milk type, intubation status, feeding frequency, and fluid requirements. The calculator automatically generates the calorie requirements and converts this into goal volume according to the particular milk type’s calorie–volume ratio. When a physician’s prescription of maintenance fluid and calorie-based protocol’s recommended feeding volume differs, the feed calculator generates the final feed volume based on the patient’s weight and type of milk. Where appropriate, water flushes were given to make up for fluid shortages when a physician’s prescribed fluid volume exceeds the recommended calorie intake to prevent calorie overfeeding.

On the other hand, when the recommended feeding volume by the calorie-based protocol exceeds the physician’s prescribed fluid volume, the latter takes precedence. Patients with over- or underfeeding would require a dietitian referral in both protocols. Lastly, the feed calculator computed the feeding volume and intravenous drip’s infusion rates at each phase of feeding increase. Nurses beta-tested the calculator on PICU patients to ensure its robustness and password protected it to prevent formula tampering before being installed in work stations in the PICU. We updated the medical team on this new calorie-based protocol and feed calculator, and they were encouraged to use the protocol. All nurses in PICU were trained and assessed competent before the calorie-based protocol officially replaced the fluid-based protocol.

### Objectives and Outcome Measures

The main objectives of this study were to assess the effectiveness of the nurse-led calorie-based protocol and nurses’ satisfaction with the use of the electronic feeds calculator. Using prospective data for the calorie-based protocol and retrospective chart review for the fluid-based protocol, we determined that our primary outcome measure was the appropriateness of calorie prescription (defined as 90%–110% of calculated energy requirements).^11^ Our secondary outcomes were (1) time taken to attain full enteral feeding from feeding initiation; (2) adequacy of calorie delivered on day 1 of full enteral feeding (defined as 80%–120% of calculated energy goal)^11^; and (3) presence of symptoms of feeding intolerance. We recorded clinical variables of the patient, such as type of and reason for admission, age, weight, the severity of illness using the pediatric index of mortality 2 score, the length of PICU stay, and duration of mechanical ventilation (MV).

To determine nurses’ satisfaction, we invited nurses with >6 months’ experience in the PICU to respond anonymously to a survey regarding the calorie-based protocol (July 2017). Nurses completed a 16-question survey that asked them to rate the protocol’s ease of use (7 questions) and quality of care in association with the protocol (6 questions) on a 1−5 Likert-type scale. The next 3 questions asked nurses to rate their comfort, confidence, and familiarity with the protocol on a 1−10 Likert-type scale. For all 16 items, lower scores denote poorer outcomes. Demographics such as the nurses’ job classification, years of experience in PICU, and the highest level of education were collected.

### Sample Size

The prospective data collection for the calorie-based protocol was planned to continue until we attained a similar sample size as the fluid-based protocol.

### Statistical Method

All statistical analysis was performed using SPSS (IBM SPSS Statistics for Windows, Version 19.0, Armonk, NY, USA), and statistical significance was taken as *P* <0.05. Pearson χ^2^ was used to test the independence of demographic and clinical characteristics with the enrolment into each protocol. Continuous variables were presented in medians/interquartile ranges (IQRs). Adequacy of calorie prescription and delivery and symptoms of feeding intolerance were presented as frequencies. The outcomes in the calorie-based and fluid-based protocols were analyzed using Mann–Whitney U tests and Pearson’s χ^2^ for continuous and frequency data, respectively. We performed subgroup analysis for patients with 3 and 4 hourly feedings as they are expected to achieve full enteral feedings at different times because of their feeding intervals. For the survey on nursing staff, the reliability of domains was analyzed using Cronbach’s alpha and ratings presented in medians/IQRs.

## RESULTS

### Baseline Characteristics

A total of 125 and 137 patients were enrolled in the fluid- and calorie-based protocols, respectively. Of these patients, 50 and 45 patients, in the fluid-based and calorie-based protocols, respectively, were excluded from analyses (see Figure, Supplemental Digital Content 5, which illustrates the flow of patient recruitment, http://links.lww.com/PQ9/A154). Thirty-one were repeat patients with multiple admissions, 49 initiated feeding before the use of any protocol, 5 used multiple milk formulae, 1 had unclear charting, and 1 patient was older than 18 years old. Also, patients started on continuous feeds were also excluded from our analysis as the patients recruited on fluid based (n = 3) and calorie based (n = 5) were too few for meaningful analysis. Seventy-five and 92 patients remained, of which 45 (60.0%) and 59 (64.1%) were fed 3 hourly in the fluid- and calorie-based protocols, respectively.

The overall median age of patients was 14 months (IQR = 4−49) (Table 1). The most common reasons for PICU admission were respiratory (n = 77, 46.1%), followed by neurological (n = 55, 32.9%) and ear-nose-throat (n = 12, 7.2%). The median length of the PICU stay was 4.7 days (IQR = 2.6−8.9), and the duration of MV was 2.2 days (IQR = 0−5.9). The median PIM2 risk of mortality was 3.06% (IQR = 1.11 - 7.40). There was no significant difference between the 2 groups on any of these characteristics.

**Table 1.**
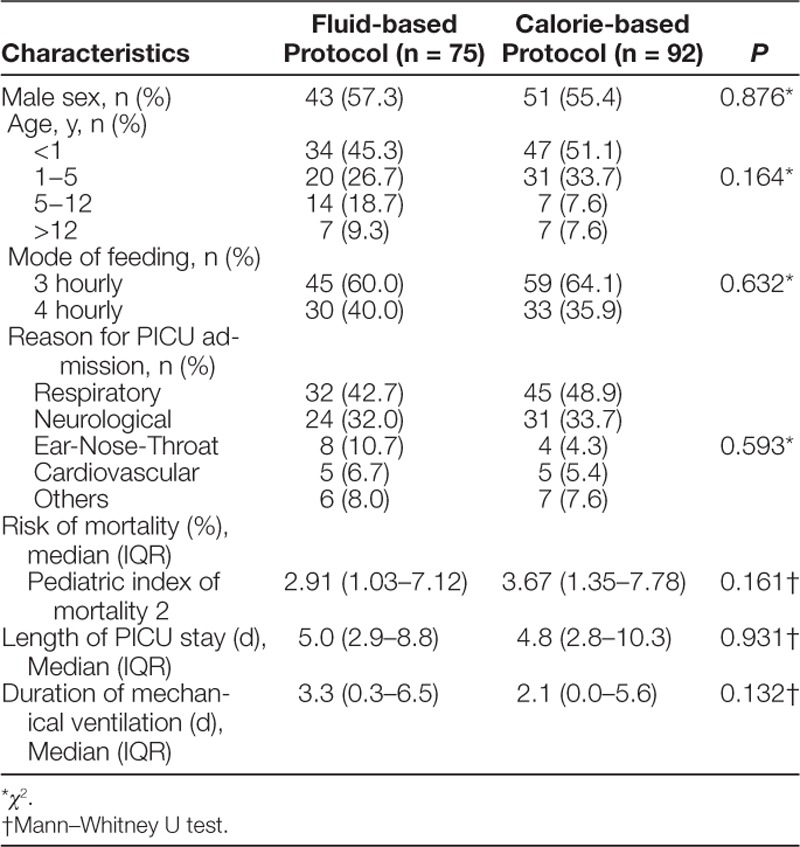
Clinical Characteristics

### Calorie Prescription and Delivery

There was a difference in the proportion of patients in each of the calorie-prescription category for the calorie- and fluid-based protocols (*P* = 0.002) (Table 2). For the assessment of calories delivered on day 1 of full enteral feeding, only 38 of 75 (50.7%) and 63 of 92 (68.5%) patients in the fluid- and calorie-based protocol, respectively, remained in the ICU and did not convert to an oral diet. Nonsignificant changes were seen in proportion of patients delivered appropriate feeds (42.1% versus 52.4%), overfed (18.4% versus 4.8%), and underfed (39.5% versus 42.9%) (*P* = 0.080) (Table 2).

**Table 2.**
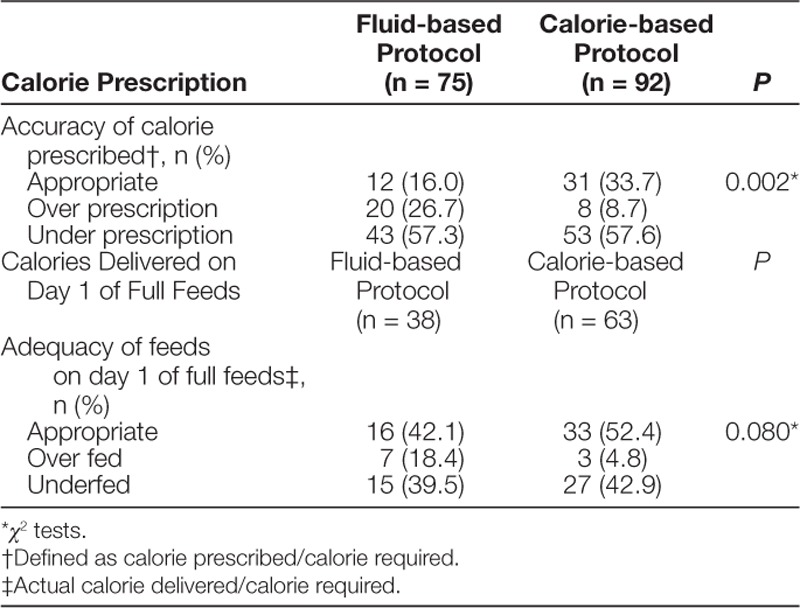
Calorie Prescription and Delivery

### Timeliness in Provision of Feeds

The time required to attain full enteral feeding significantly improved with the calorie-based protocol. Median time taken from protocol initiation to full feeds (18.0 hours, IQR = 18.0−27.5 versus 12.8 hours, IQR = 12.0−16.0, *P* < 0.001) and time of PICU admission to full feeds (43.0 hours, IQR = 33.5−62.5 versus 35.9 hours, IQR = 24.0−48.7, *P* = 0.021) significantly reduced in patients on the caloric-based protocol compared with fluid-based protocol (see Table, Supplemental Digital Content 6, which compares time taken to achieve goal feeds and feeding intolerance rates between the 2 protocols, http://links.lww.com/PQ9/A154). There was no difference in the time required to initiate feeds (median: 20.0 hours, IQR = 9.0−36.5 versus 18.3 hours, IQR = 11.0−34.4, *P* = 0.292). The majority of patients in both protocols did not experience symptoms of feeding intolerance (78.7% versus 83.7%, *P* = 0.604). The symptoms of feeding intolerance included high gastric residual volume (16.0% versus 10.9%), abdominal distension and vomiting (5.3% versus 4.3%), and others (0% versus 1.1%) in the fluid- and calorie-based protocols, respectively.

**Fig. 2.**
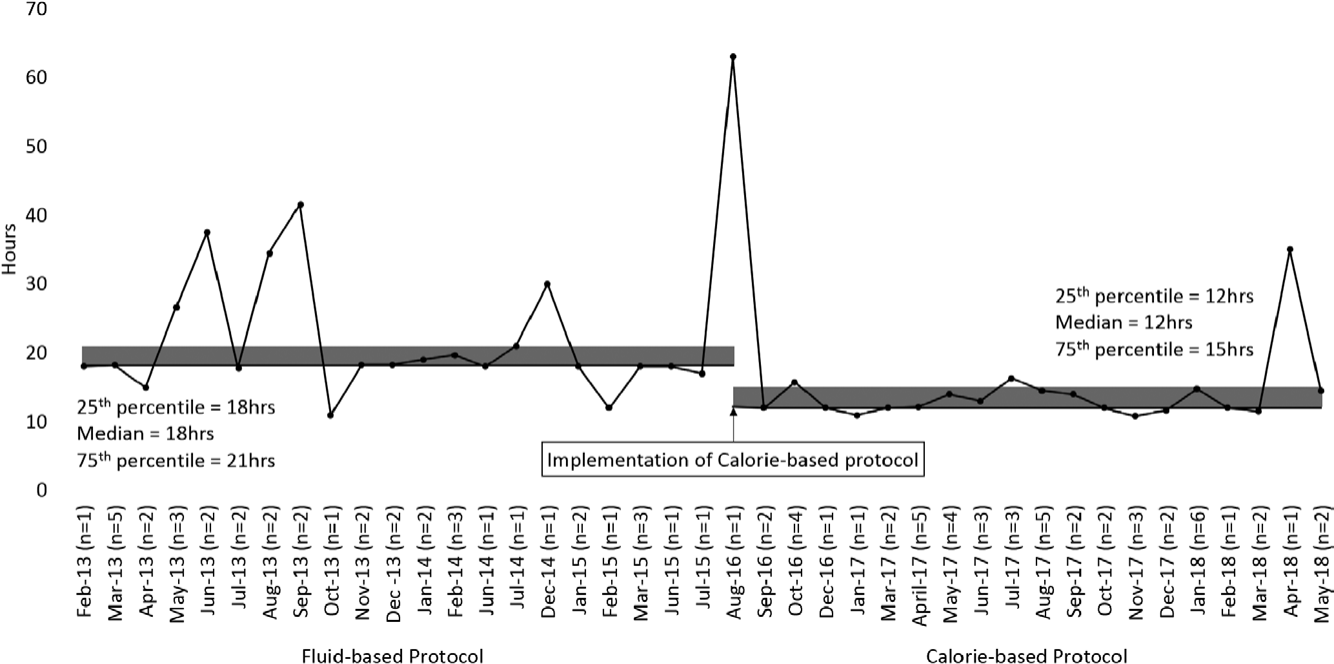
Time taken to reach full feeds (3 hourly).

**Fig. 3.**
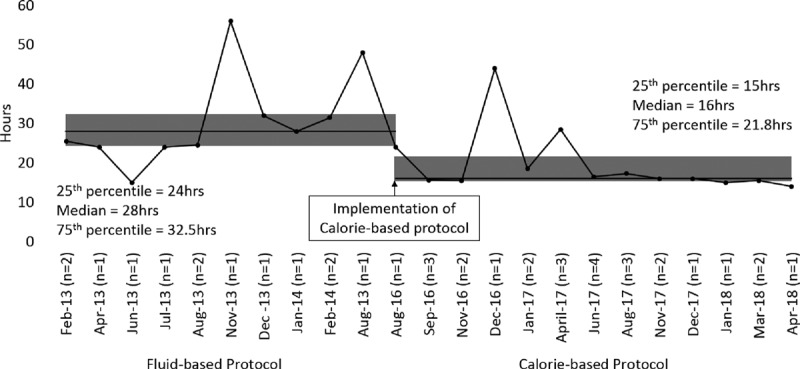
Time taken to reach full feeds (4 hourly).

We performed a subgroup analysis for patients with 3 hourly and 4 hourly feedings (Table 3). In patients with 3 hourly feedings, the time to attain full enteral feeding from protocol initiation was reduced from 18.0 hours (IQR = 18.0−21.0) in the fluid based to 12.0 hours (IQR = 12.0−15.0) in the calorie-based protocol (*P* < 0.001) (Fig. 2). Among patients with 4 hourly feedings, the reduction in time was from 28.0 hours (IQR = 24.0−32.5) in the fluid-based protocol to 16.0 hours (IQR = 15.0−21.8) in the calorie-based protocol (*P* = 0.001) (Fig. 3). There was no difference in the time from PICU admission to initiation of both protocols: 19.5 hours (IQR = 9.0−33.8) to 14.9 hours (IQR = 9.5−24.7) in the 3 hourly feeding group (*P* = 0.256) and 21.0 hours (IQR = 13.5−42.0) to 33.8 hours (IQR = 17.8−49.1) in the 4 hourly feeding group (*P* = 0.940). As a result, a significantly shorter amount of time was taken to attain full enteral feeding from PICU admission for the calorie-based protocol (median: 29.9 hours, IQR = 23.0−40.0) compared with the fluid-based protocol (median: 39.5 hours, IQR = 27.5−59.0) in the 3 hourly feeding group (*P* = 0.002). A nonsignificant reduction is seen in the 4 hourly feeding group (*P* = 0.655) from 59.0 hours (IQR = 38.0−69.0) in the fluid-based protocol to 49.3 hours (IQR = 35.2−68.0) in the calorie-based protocol.

**Table 3.**
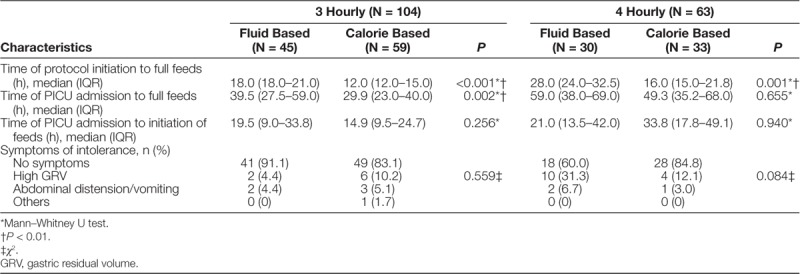
Outcome Measures by Feeding Frequencies

### Nursing Perception

Sixty-three (77%) nurses responded to the survey regarding the calorie-based protocol (see Table, Supplemental Digital Content 7, which illustrates nursing perceptions toward the calorie-based protocol, http://links.lww.com/PQ9/A154). There was good internal consistency for Ease of Use and Quality of Care survey components (Cronbach’s alpha *r* = 0.793 and *r* = 0.915, respectively). On a Likert Scale of 1−5, nurses perceived the calorie-based protocol to be easy to use (3.9, IQR = 3.6−4.1) and improved quality of care (4.0, IQR = 3.5−4.0). Assessed with a 1−10 scale, nurses were also comfortable (7.0, IQR = 7.0−9.0), confident (8.0, IQR = 7.0−9.0), and familiar (8.0, IQR = 7.0−9.0) with the new protocol.

## DISCUSSION

Our study demonstrated the acceptability of tailoring feeding prescription to individual calorie and fluid requirements in a nurse-led EN protocol. The calorie-based protocol increased the proportion of patients prescribed with appropriate calories. The main impact was seen in the reduction in the overprescription of calories in feeds. The time required to attain full enteral feedings from feed initiation decreased without an increase in feeding intolerance. We saw no significant changes in the proportion of patients receiving appropriate calories on day 1 of full enteral feeding.

Our results showed that the addition of the calorie-based protocol significantly reduced the overprescription of feeds. Most published EN protocols defined energy goals using formulas by WHO, Schofield’s, White, or Estimated Average Requirements.^12–18^ Pediatric energy expenditure varies among patients depending on their age, anthropometry, and clinical variables. Published protocols in the pediatric intensive care literature derive feeding goals based on caloric requirements specified by dietitians,^13^ nutrition teams,^12,19^ or attending physicians.^18,20^ However, PICUs without nutrition expertise in the form of dedicated dietitians or nutrition teams may not be able to establish feasible and sustainable caloric goals.^21^ The applicability of existing protocols may be limited to resource-rich PICUs. It is thus unsurprising that in some PICUs, maintenance fluids are converted into enteral feeding goals as per the fluid-based protocol in the “before” period of our study. Our results highlighted that the routine conversion of pediatric maintenance fluid to enteral feeds systematically overfed a significant proportion of PICU patients.

In our study, improvement in the accuracy of calorie prescription did not translate to improvements in actual calorie delivery. The discrepancy between calorie prescription and delivery has similarly been described in other PICUs.^18,22^ Gastrointestinal feeding intolerance and various procedures such as extubation, surgical interventions, and scans often interrupt the delivery of EN.^17,18,20,22^ Despite these interruptions, de Neef et al^20^ found the discrepancy of delivery and prescription to be statistically insignificant and small. Their findings may be related to their exclusive use of the postpyloric route, strategies for maintenance of EN in cases of planned procedures, or patient profile.^22^ Exacerbating the issue of calorie deficits is the prescription of insufficient calories in our study, most often secondary to fluid restriction. Other studies have also found factors such as patients’ clinical conditions, providers’ medical and EN knowledge, and support of the nutrition team to affect EN prescription.^17,18^

The early delivery of nutrition is associated with lower mortality rates in observational studies.^5,23^ Nutritional algorithms in ICUs support an automated process for feeding initiation and advancement and delineated actions for feeding intolerance. The calorie-based protocol advanced feeds at a faster rate of 25%–25%–50%–75%–100% compared with 25%–25%–50%–50%–75%–75%–100% in the original fluid-based protocol. Full enteral feeding was attained within a short time while not increasing the rate of gastrointestinal intolerance. The success of feeding protocols in achieving goal feeds within a shorter time has similarly been shown in other PICUs despite their use of different progression rates and continuous mode of feeding.^12–15^ Although the calorie-based protocol did not improve the time required to initiate feeds, the overall time taken to achieve full enteral feeding from PICU admission was significantly improved. Both indicators are in line with suggestions by the Society of Critical Care Medicine-American Society for Parenteral and Enteral Nutrition guidelines to initiate feeds within 24−48 hours of PICU admission and to deliver up to two-thirds of the feeding goals within 1 week of critical illness.^8^ Similar to other studies, we did not find a statistically significant improvement in the PICU length of stay or duration of MV with the appropriate caloric prescription of feeds or faster achievement of full feeds.^13–15,24^

In our improvement project, manual calculation on pen and paper was converted to the electronic feeds calculator, which integrated the stepwise EN algorithm. Nurses perceived the calorie-based protocol to be easy to use, improved quality of care, and they were comfortable with it. Voluntary strategies with a focus on nurses’ intrinsic motivation were used in the implementation.^25^ Training sessions imparted knowledge about relevant aspects of nutrition management and the use of the electronic feeds calculator. We provided continuous support to the nurses and sought their feedback. The use of nurse-led protocols in weaning of MV, management of sedation, and EN has become more prevalent in ICUs today.^26–28^ A qualitative study sampled 5 National Health Service sites in the United Kingdom which utilized standardized care approaches and similarly found favorable attitudes.^29^ Among 141 participants, 73 were nurses recruited from a cardiac surgical unit, walk-in health center, preoperative assessment clinics, midwife-led birth center, and a General Practice Surgery service. Nurses favored standardized care for it allows extension of nursing roles and supports autonomy in nursing practice.^25^

There are limitations to our study. First, we conducted our study in a single center in Singapore, which resulted in a small sample size. Also, due to the conservative criteria of our feeding protocol, our patients had lower Pediatric Index of Mortality scores compared with other studies.^14,18,20^ The results of this study may only be generalizable to other centers with similar characteristics. Also, we did not include the results of patients fed continuously and those patients receiving multiple formulas. Future studies need to be performed on patients with continuous feeding and receiving multiple formulas to evaluate the effectiveness of our proposed calorie-based protocol. Furthermore, our findings on patients on 3 and 4 hourly feedings should be validated in future studies. For this study, as there was no available survey to measure nursing satisfaction with a nurse-led feeding protocol, we developed a survey specifically to capture nursing satisfaction quantitatively. However, this survey was not validated. Nevertheless, our simple survey showed that the majority of the nurses were satisfied with the new protocol. Lastly, we recognize that predictive equations remain inaccurate compared with indirect calorimetry (IC). IC, while recognized as the gold standard in measuring resting energy expenditure during critical illness, is not readily available in most PICUs due to lack of resources or expertise. In the absence of IC, predictive equations remain a viable option^8^ and are preferable to using fluid maintenance requirements.

## CONCLUSIONS

The use of a calorie-based EN protocol that incorporated an electronic feeds calculator was well accepted by nurses in our PICU and led to significant improvements in the accuracy of feeds prescription, and time required to attain full enteral feeding from feeds initiation and PICU admission. PICUs without dedicated dietitians or nutrition teams can consider the use of a simple electronic feeds calculator. Our quality improvement study highlighted 2 pervasive issues related to calorie deficit: inadequate delivery due to feeding interruption and the under prescription of calories. Future studies should investigate strategies to address these 2 important areas of pediatric critical care nutrition management.

## ACKNOWLEDGMENTS

Assistance with the study was provided pediatric intensive care units (PICU) nurses and physicians for supporting the implementation of the feeding protocol in their daily care of patients. Sam Koh (Office of Quality, Safety, and Risk Management) assisted in the planning phase when the protocol was initially implemented in the PICU and during the data analysis for this manuscript.

## DISCLOSURE

The authors have no financial interest to declare in relation to the content of this article.

## Supplementary Material

SUPPLEMENTARY MATERIAL
