# Supplementary material for: Use of an Electronic Feeds Calorie Calculator in the Pediatric Intensive Care Unit
Source: Pediatr Qual Saf. 2020 Jan 12;5(1):e249. doi: 10.1097/pq9.0000000000000249 (PMC7056286; doi:10.1097/pq9.0000000000000249)
Supplement: SUPPLEMENTARY MATERIAL [file pqs-5-e249-s001.pdf]

Supplemental Digital Content 1.
Table: Calories provided by Fluid-based and Calorie-based protocols

**Weight (kg)**	**Feed type**	**Fluid-based Protocol (kcal/kg/day)** *Based on Holliday-Segar method	**Calorie-based Protocol (kcal/kg/day)** *Based on energy requirements calculated using Schofield equation and Dietary Reference Intakes	
		**Any intubation Status**	**Intubated**	**Non-intubated**
3 – 5	For <1 year old, use EBM / Infant formula / Follow-on formula (0.68kcal/ml), including: Neosure, Alfare, Neocate, Monogen, Enfamil AR	68	70 – 85	85 – 105
6 – 10			55 – 70	70 - 85
11 – 15		55 – 65	55 – 65	70 – 80
10	For ≥1 year old, use nutritionally complete formula appropriate for age (1kcal/ml), including: Pediasure, Nutren Jr, Ensure, Isocal, Peptamen Jr	100	55 – 70	70 - 85
11 – 15		85 – 95	55 – 65	70 – 80
16 – 20		75 – 80	50 – 60	60 – 70
21 – 25		65 – 70	40 – 50	55 – 60
26 – 30		55 – 65	40 – 45	50 – 55
31 – 40		45 – 55	35 – 45	40 – 50
41 – 50		40 – 50	30 – 40	35 – 45
51 – 60		40 – 45	30 – 35	30 – 40
>60		35 – 40	25 – 30	30 – 35
11 – 15	For >1 year old who requires growing-up formula	65 – 70	55 – 65	70 – 80
16 – 20		55 – 60	50 – 60	60 – 70
21 – 25		50 – 55	40 – 50	55 – 60
