# Supplementary material for: Use of an Electronic Feeds Calorie Calculator in the Pediatric Intensive Care Unit
Source: Pediatr Qual Saf. 2020 Jan 12;5(1):e249. doi: 10.1097/pq9.0000000000000249 (PMC7056286; doi:10.1097/pq9.0000000000000249)
Supplement: SUPPLEMENTARY MATERIAL [file pqs-5-e249-s002.pdf]

Supplemental Digital Content 2.
Table: Schofield equations for estimating basal metabolic rate from weight.

	Basal metabolic rate (kcal/24 hours)
Children under 3 years of age	
Male	59.5 x weight [kg] – 30.4
Female	58.3 x weight [kg] – 31.1
Children 3 to 10 years	
Male	22.7 x weight [kg] + 504.3
Female	20.3 x weight [kg] + 485.9
Children 10 to 18 years	
Male	17.7 x weight [kg] + 658.2
Female	13.4 x weight [kg] + 692.6

Reference: Schofield W. Predicting basal metabolic rate, new standards and review of previous work. *Human nutrition Clinical nutrition.* 1985;39:5-41.
