# Supplementary material for: Use of an Electronic Feeds Calorie Calculator in the Pediatric Intensive Care Unit
Source: Pediatr Qual Saf. 2020 Jan 12;5(1):e249. doi: 10.1097/pq9.0000000000000249 (PMC7056286; doi:10.1097/pq9.0000000000000249)
Supplement: SUPPLEMENTARY MATERIAL [file pqs-5-e249-s003.pdf]

Supplemental Digital Content 3

Figure: Calorie-based protocol algorithm

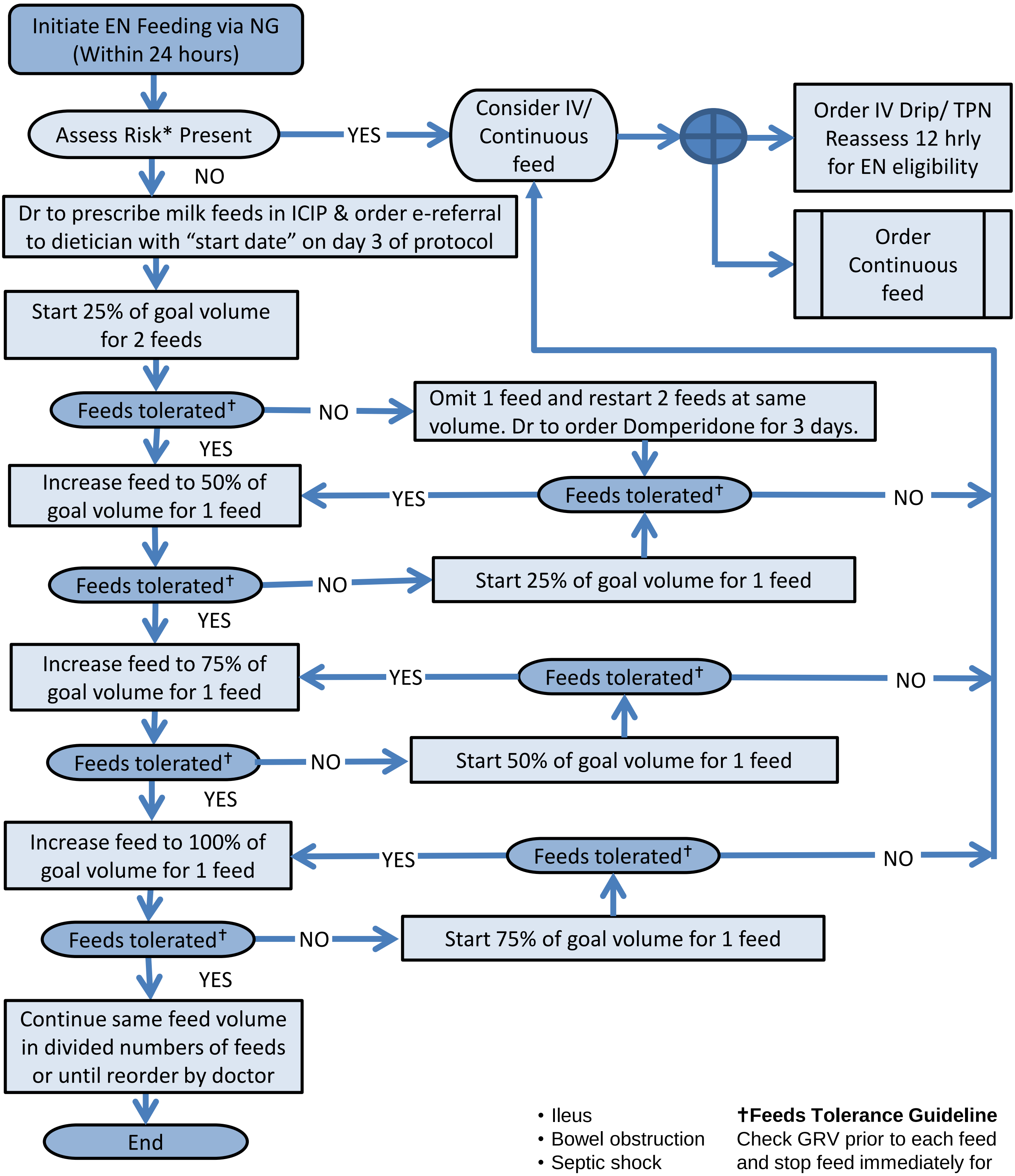

*__Assess RISK present__
- High Gastric Losses
- Abdomen distension
- Significant GI bleed
- Complex GI surgery
- Entericanastomosis
- Central cooling
- Enteric fistula
- Intractable diarrhoea
- Severe exacerbation of inflammatory bowel
- Awaiting extubation within 6 hours.
- Patients on mixed feeds (i.e. 2 or more different formula
- Ileus
- Bowel obstruction
- Septic shock
- Post cardiac surgery
- Open chest
- NEC
- Unstable respiratory status in a non-intubated patient

†Feeds Tolerance Guideline

Check GRV prior to each feed and stop feed immediately for presence of any condition below:
- High GRV> ½ previous feed volume
- Increased abdominal distension
- Presence of nausea, vomiting, diarrhoea
