# Supplementary material for: Use of an Electronic Feeds Calorie Calculator in the Pediatric Intensive Care Unit
Source: Pediatr Qual Saf. 2020 Jan 12;5(1):e249. doi: 10.1097/pq9.0000000000000249 (PMC7056286; doi:10.1097/pq9.0000000000000249)
Supplement: SUPPLEMENTARY MATERIAL [file pqs-5-e249-s004.pdf]

Supplemental Digital Content 4. Electronic Feeds Calculator

KK Women's and Children's Hospital
SingHealth

Schedule for Intermittent Feeds

see tab below for continuous feed

Patient name label

Date:	23/9/2019
Bed No:	1
Intubation status:	Intubated

Weight (kg): **10.0**

No. of feeds/day: **8**

Total fluid (ml/d):	1000
Amt substrate for IV Med (ml):	0
Remaining fluids (ml):	1000
Milk type:	EBM
Milk given (ml/day):	1000 — **OK**
Milk required (ml/day):	830 to 1003
Start up drip rate (ml/h):	42
Target volume (ml/feed):	125

Date/ Time to be given	**Gastric Residual Volume Feed Tolerance** Check GRV before each feed; Stop feed immediately if following condition is present:	**Feeds** % Feed	Milk (ml)	Water bolus (ml)	Total feed (ml)	**Drip** % Drip	Drip rate (ml/hr)	**Remarks**
	☐ GRV >1/2 previous feed vol:_____ml<br>☐ Increased abdominal distention<br>☐ Nausea, vomiting, diarrhoea	25%	**30**	**0**	30	75%	31	
	☐ GRV >1/2 previous feed vol:_____ml<br>☐ Increased abdominal distention<br>☐ Nausea, vomiting, diarrhoea	25%	**30**	**0**	30	75%	31	
	☐ GRV >1/2 previous feed vol:_____ml<br>☐ Increased abdominal distention<br>☐ Nausea, vomiting, diarrhoea	50%	**65**	**0**	65	50%	21	
	☐ GRV >1/2 previous feed vol:_____ml<br>☐ Increased abdominal distention<br>☐ Nausea, vomiting, diarrhoea	75%	**95**	**0**	95	25%	10	
	☐ GRV >1/2 previous feed vol:_____ml<br>☐ Increased abdominal distention<br>☐ Nausea, vomiting, diarrhoea	100%	**125**	**0**	125	0%	0	

Scheduled by:_________________ Signature:_________________

Counter check by:_______________ Signature:_________________
