# Supplementary figures and images for: Use of an Electronic Feeds Calorie Calculator in the Pediatric Intensive Care Unit
Source: Pediatr Qual Saf. 2020 Jan 12;5(1):e249. doi: 10.1097/pq9.0000000000000249 (PMC7056286; doi:10.1097/pq9.0000000000000249)

## Supplemental Digital Content 5

Figure: Flow Diagram of Patient Enrolment

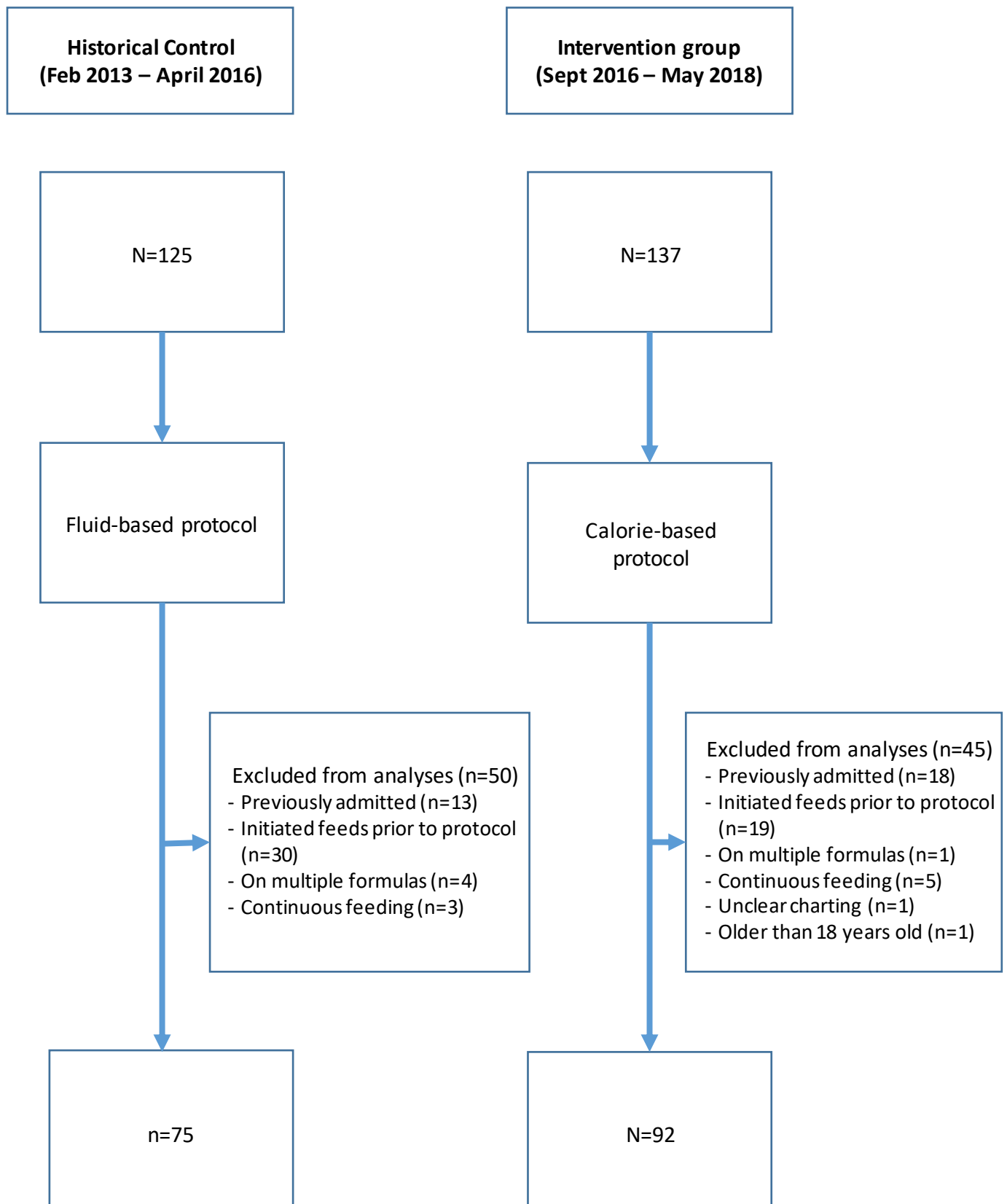

Supplement: SUPPLEMENTARY MATERIAL [file pqs-5-e249-s005.pdf]
