# Supplementary material for: Use of an Electronic Feeds Calorie Calculator in the Pediatric Intensive Care Unit
Source: Pediatr Qual Saf. 2020 Jan 12;5(1):e249. doi: 10.1097/pq9.0000000000000249 (PMC7056286; doi:10.1097/pq9.0000000000000249)
Supplement: SUPPLEMENTARY MATERIAL [file pqs-5-e249-s006.pdf]

Supplemental Digital Content 6.

Table: Timeliness of enteral feeds

Characteristics	Fluid-based protocol (N=75)	Calorie-based protocol (N=92)	p-value
Time of PICU admission to initiation of feeds (hours), median (IQR)	20.0 (9.0, 36.5)	18.3 (11.0, 34.4)	0.292[b]
Time of protocol initiation to full feeds (hours), median (IQR)	18.0 (18.0, 27.5)[c]	12.8 (12.0, 16.0)[d]	<0.001[b]**
Time of PICU admission to full feeds (hours), median (IQR)	43 (33.5, 62.5)[c]	35.9 (24.0, 48.7)[d]	0.021[b]*
Symptoms of intolerance, n(%)			0.604[a]
Nil	59 (78.7)	77 (83.7)	
High GRV	12 (16.0)	10 (10.9)	
Abdominal Distension/Vomiting	4 (5.3)	4 (4.3)	
Others	0	1 (1.1)	

[a] Chi-square

[b] Mann Whitney U Test

[c] n=53 patients achieved full feeds in Fluid-based protocol

[d] n=78 patients achieved full feeds in Calorie-based protocol

*p<0.05

**p<0.01

GRV: Gastric Residual Volume

IQR: Interquartile range
