# Supplementary material for: Use of an Electronic Feeds Calorie Calculator in the Pediatric Intensive Care Unit
Source: Pediatr Qual Saf. 2020 Jan 12;5(1):e249. doi: 10.1097/pq9.0000000000000249 (PMC7056286; doi:10.1097/pq9.0000000000000249)
Supplement: SUPPLEMENTARY MATERIAL [file pqs-5-e249-s007.pdf]

Supplemental Digital Content 7.
Table: Nurses' Perceptions of the Calorie-based Protocol (N=63)

	**Median (IQR)**
**Ease of Use[a] (7 items)**	3.9 (3.6, 4.1)
**Quality of Care[a] (6 items)**	4.0 (3.5, 4.0)
**Comfort[b]**	8.0 (7.0, 9.0)
**Confidence[b]**	8.0 (7.0, 9.0)
**Familiarity[b]**	8.0 (7.0, 9.0)

[a] Measured on a 1-5 Likert Scale

[b] Measured on a 1-10 Likert Scale
